# Firewood, smoke and respiratory diseases in developing countries—The neglected role of outdoor cooking

**DOI:** 10.1371/journal.pone.0178631

**Published:** 2017-06-28

**Authors:** Jörg Langbein

**Affiliations:** 1 RWI – Leibniz Institute for Economic Research; Hohenzollernstrasse 1-3, 45128 Essen, Germany; 2 Ruhr-Universität Bochum; Universitätsstraße 150, 44801 Bochum, Germany; Liverpool School of Tropical Medicine, UNITED KINGDOM

## Abstract

Smoke from cooking in the kitchen is one of the world’s leading causes of premature child death, claiming the lives of 500,000 children under five annually. This study analyses the role of outdoor cooking and the prevalence of respiratory diseases among children under five years by means of probit regressions using information from 41 surveys conducted in 30 developing countries from Asia, Africa and Latin America. I find that outdoor cooking reduces respiratory diseases among young children aged 0-4 by around 9 percent, an effect that reaches 13 percent among children aged 0-1. The results suggest that simple behavioral interventions, such as promoting outdoor cooking, can have a substantial impact on health hazards.

## 1 Introduction

About 3 billion people in developing countries rely on firewood or charcoal for their daily cooking purposes [1]. According to the World Health Organization (WHO), smoke-induced diseases are responsible for the death of 4.3 million people every year—more deaths than caused by malaria or tuberculosis—making it one of the most lethal environmental health risks worldwide [2, 3]. The largest burden of mortality is borne by women and young children. Among the 4.3 million who die from the consequences of smoke emission each year, 500,000 are children under five that die due to acute respiratory infections (ARI).

Young children are particularly vulnerable for two reasons: First, they are usually with their mothers during the cooking process and thus inhale large loads of particulate emission. In a recent systematic review, it was found that childrens’ particulate emission exposure is similar to their mothers’ [4]. Second, in comparison to adults, the still growing bodies of young children are more susceptible to ARI, leading to a high death rate in this age group [5].

One proposed remedy are clean cookstoves, such as LPG stoves. However, these cookstoves are expensive and not widespread. Even their more affordable cousin, improved cooking stoves (ICS), which tend to have fewer health and environmental benefits, are only used by around one third of the 2.85 billion people who rely on solid fuel [6]. In response, many have advocated behavioral change interventions targeting cooking behavior as low-cost interventions to reduce the detrimental health effects from emitted smoke exposure [7–9]. Such interventions include health education and knowledge dissemination, cooking appliance maintenance training or information on ventilation practices. Yet another behavioral change is the setting of the cooking location, which is the focus of this paper. Cooking outdoors instead of indoors may have important implications for ventilation and thus smoke exposure [10–12], but has so far been widely neglected in the debate. A review of studies published from 1983 to 2013 suggests that behavioral changes alone may reduce indoor air pollution exposure by 31 to 85 percent [5]. The largest reduction of indoor air pollution exposure was found in a rigorous field study in China, where health education, behavioral activities, and a cookstove intervention were combined [13].

This paper explores the correlation of outdoor cooking with acute respiratory infections of children under 5 who live in rural areas of 30 developing countries spanning Asia, Africa and Latin America. So far, only a few articles have explicitly addressed the impact of the cooking location on particulate matter exposure [14, 15] and respiratory diseases [16–19]. All of these studies cover only one geographical area of a country and/or use a small dataset. Rehfuess et al. (2009) and Buchner and Rehfuess (2015) are notable exceptions, reporting the effect of outdoor cooking on health for 16 and 9 Sub-Saharan countries [18, 19].

The source of the present data are the Demographic Health Surveys (DHS), regular cross-sectional surveys in developing countries with a focus on health issues. This study examines 41 surveys conducted in 30 countries, yielding 219,776 observations from the years 2005 to 2014. The decision to cook outdoors is likely jointly determined by other factors. Examples include but are not limited to education, income, cultural factors or climate. This gives rise to potential endogeneity issues, which precludes ascribing a causal interpretation to the effect of outdoor cooking. In order to reduce the omitted variable bias arising from endogeneity, first a probit model is estimated that controls for confounding variables. In a second step, the probit estimations are subject to several robustness checks, including a matching approach and controlling for smoking behavior. Results are presented on an aggregated level over all countries as well as on a single country level, since cultural differences are one potential source of bias. In order to account for younger children being more susceptible to ARI than older ones, I estimate in a separate model the effect of outdoor cooking on health for children aged 0-1.

It is found that outdoor cooking is associated with a decrease of 0.5 percentage points in the ARI occurrence of children aged 0-4 and with a decrease of 1 percentage point for children aged 0-1 compared to indoor cooking. These coefficients correspond to a reduction due to outdoor cooking of 9 percent of all ARI occurrences for children aged 0-4 and to 13 percent for children aged 0-1. The relationship is significant and remains stable when including control variables or conducting several robustness checks.

The paper is structured as follows: Section 2 describes the literature and some theoretical predictions derived from it. Section 3 provides information on the dataset. The estimation strategy is discussed in Section 4 and results are presented in Section 5. Section 6 concludes.

## 2 Literature and theoretical prediction

The negative impact of air pollution on health has increased over the last 20 years [20], as has the documentation of it. Combusting solid fuel in-house affects health in various ways and may contribute to acute respiratory infections, stunted growth in children, pneumonia, chronic bronchitis in women, chronic obstructive pulmonary disease (COPD), cataracts and other visual impairments, cardiovascular diseases, lung cancer, tuberculosis, and perinatal diseases [21–27]. Estimates from the WHO (2014) suggest that the exposure to household air pollution from cooking with solid fuels causes 4.3 million premature deaths annually [2]. The potential harm does not stop once the smoke leaves the house, leading to another 0.5 million premature deaths in 2010 due to ambient air pollution exposure [28–30]. Several studies highlight that a child’s body is more susceptible to ARI due to still developing lungs and immune systems, which is exacerbated by higher respiration rates among children that lead to around 50 percent more of the polluted air breathed in compared to adults [31–33].

Key foci of the literature on cookstoves have been on ventilation practices as well as the effect of ambient air pollution on indoor air pollution [15, 34, 35]. In that context, regular ventilation is generally thought to decrease the particulate matter concentration in the kitchen and other rooms in the house. The same rationale goes for outdoor cooking, where particulate matter directly dissipates in the surroundings. Other studies explicitly analyse the particulate matter concentration for 24 hours on a small scale for indoors and outdoors. Although those studies report a large drop in the concentration level by cooking outside, they note that the level found is still larger than the recommended level by the WHO [36–38]. Among the few papers that have explicitly focused on outdoor cooking and health outcomes, Albalak et al. (1999) compare two villages in Bolivia, one cooking outdoors and one indoors [16]. They find that particulate matter exposure is twice as high in households in the indoor cooking village compared to the outdoor cooking village, translating to a bronchitis reduction of 60 percent. Similar results are found by Owusu and Kuitunen (2006) for Accra, who highlight a stark correlation between children’s presence in the indoor cooking area and respiratory diseases [17]. Rehfuess et al. (2009) conclude in a cross country study among 16 African countries that the impact of cooking with solids fuels on acute lower respiratory infections is different among children with regards to ventilation practices and the cooking location [18]. Higher ventilation and outdoor cooking decreases the risk of ARI. Buchner and Rehfuess (2015) obtain similar results when analyzing the cooking location in 9 Sub-Saharan African countries [19]. Both studies report average impacts over all countries.

This paper adds to the existing literature by analyzing the issue using a large sample from different countries, thus, yielding broad geographical coverage. The focus is on the link between the cooking location and incidence of infection, controlling for potential confounding factors.

## 3 Data

I use data from the Demographic Health Surveys (DHS) to assess the relationship between outdoor cooking and health. The DHS are nationally representative, cross-sectional household surveys with a main survey that focuses on general health conditions and demographics and complementary surveys focusing on explicit health topics, such as malaria and HIV.

All countries included in this analysis are low-income or lower middle-income countries in Africa, Latin America, and South-East Asia according to the World Bank classifications as of October 31, 2016. Due to data regulations, lack of data collection and lack of variation in outdoor cooking, only 30 of 60 countries fit this classification and are included in the analysis, of which 27 are in Africa, one in Asia and two in Latin America. Necessary information on the cooking locations is found in wave 5, wave 6 and wave 7. The analysis is restricted to the main DHS and does not include any other specifically framed questionnaires. Included countries had to meet a threshold of outdoor cooking of at least 10 percent.

Assuming that ambient air pollution is more prevalent in urban regions, I focus on rural regions to reduce this bias. Another reason for the focus on rural areas is that households should be able to decide between indoor and outdoor cooking, whereby available space may be limited in urban areas. Hence, the results only apply to households living in a rural environment and cannot be directly transferred to urban environments.

The main DHS questionnaire asks questions on stove usage, cooking fuels and cooking location but due to a massive lack of responses, the answers on stove usage could not be included in this analysis. Regarding the cooking location, households were asked whether they usually cook in the house, in a separate building, or outside. Multiple answers were not possible. In this analysis, cooking in a separate building and cooking in the house are combined and analyzed as indoor cooking, but an additional robustness check excludes cooking in a separate building, thereby focusing only on the comparison of cooking in the house and outdoors. Cooking fuels are controlled for throughout this analysis. As another robustness test on the effect of outdoor cooking, I restrict the samples to those households using firewood as cooking fuel. This should yield a more precise estimate of the impact of cooking outdoors using solid fuels because cooking with electricity and LPG, which are clean cooking fuels, is mostly done indoors.

Information on the child’s health, answered by the head of household’s wife is merged with the household dataset. The main outcome variable “ARI” is binary and constructed using information on coughing behavior of children aged 0-4 years in the household in the last two weeks before the survey was administered. Mothers were asked to report if the child aged 0-4 years had a cough in the last two weeks before the survey was administered. If this was true, the mother was asked if the coughing was accompanied with fast and heavy breathing due to a problem in the chest and/or nose. When these conditions were met, the pattern was classified as ARI by the DHS program [39].

To obtain results for this age group only, and since there is no data provided for other household members, all households without children age 0-4 years are dropped. The unit of observation are children under the age of 5 and households with several children under 5 appear multiple times in the data.

Overall, 219,776 observations from 30 countries and the years 2005 to 2014 are included. S1 Table shows the numbers of observations by country and survey year in rural areas.

## 4 Empirical strategy

Deriving a strictly causal interpretation of the relationship between the cooking location and potential impacts on children’s health in this setup is complicated by the fact that the cooking location is endogenously determined. For example, it may be that better educated household heads or the persons cooking know about the negative health effects of household air pollution and thus cook outdoors. This decision may also be correlated with the household’s earnings. Although I control for education and wealth indicators as proxies for income and similar confounders, it is not possible to rule out the existence of unobserved variables, such as astuteness or health knowledge, that bias the estimates through their correlation with the outdoor cooking decision.

To estimate the effect of outdoor cooking on the probability that a child suffers from ARI in the last two weeks before the survey, I specify a probit model, in which the binary variable ARI is regressed on the cooking location and a set of control variables.

All waves are pooled and country dummies are included in every estimation to account for country differences. A dummy for the year of the interview is also included to control for time trends. Recognizing that previous research has identified the season as an important driver of ARI, a dummy variable controlling for the occurrence of a rainy season during the time of the interview is constructed [40–42]. This was done using information on the rainy seasons from the CIA Factbook. If the needed information could not be found there, other sources were used to obtain information on the seasons. The variable also takes regional variations of the rainy seasons within the same country into account. However, the interpretation of this variable has to be treated with caution for two reasons: First, it measures whether the interview was conducted during a rainy season but the indicator I look at is retrospective and asks for the occurrence of ARI in the last two weeks before the survey. Second, the survey implementation is not independent of the rainy season and in many cases surveys were only conducted in dry season. Thus, for around half of the countries, there are no observation in both, rainy and dry season and thus, the rainy season dummy is picking up some idiosyncratic country effect. At the household level, I control for several socio-economic household characteristics, such as ownership of motorbikes, bicycles, cars and/or television, housing wall quality as well as the number of household members, and availability of piped water. An additional binary variable is constructed controlling for regular smoking within the house. The variable takes the value 1 if household members smoke daily or weekly in the house and 0 otherwise. Yet as this variable suffers from a large share of missing observations, this is only included in a robustness check. Regarding the household composition, the age of the household head, the sex of the household head and the education level of the wife are included as controls. At the child level, I control for the child’s sex, age and whether the child has ever been vaccinated. As with regular smoking, the vaccination indicator suffers from a large number of missing observations and can thus only be included in a robustness check and not in the main specification. See S2 Table for a summary of the variables and the level they are collected. Standard errors are clustered at the household level since children are nested within households.

## 5 Results

### 5.1 Descriptive results

The number of households cooking outdoors varies quite substantially across continents: In Africa, every third household cooks outside, compared to only 15 percent in Asia and 43 percent in Latin America. Due to the dominance of African countries in the data, every third household in the sample cooks outdoors. There are large disparities within Africa, with only 18 percent of the households cooking outdoors in East Africa compared to 43 percent in West Africa (see S3 Table). There are minor differences in outdoor cooking across seasons. If the interview was conducted in a dry season 38 percent of the households cooked outside, whereas this share amounted to 31 percent of the households in rainy seasons. Note that the rainy season variable describes whether the region where the household was interviewed in this month was in a rainy season month. Hence, the variable is collected on a regional level, rendering any meaningful interactions with household variables impossible. All following models are estimated with and without the rainy season dummy to check their robustness. The coefficient size and the significance levels remained the same.

Among children aged 0-4 (0-1) around 7 (8) percent suffered from acute respiratory infections in the two weeks preceding the survey. The share is different between the three continents: In African countries, on average 6 percent of the children aged 0-4 suffered from ARI indications, compared to 9 percent in the Philippines as Asian country and 19 percent in the Latin American countries. This share is slightly higher among the subgroup of children aged between 0 and 1, where 8, 9 and 20 percent of the children suffered from ARI indications in Africa, Asia and Latin America, respectively. In West Africa 4 percent (5 percent) of the children aged 0-4 (0-1) suffered from ARI compared to 10 percent (12 percent) in East Africa (all values S3 Table).

With regards to the control variables, it can be seen that around 92 percent of the included households use firewood as their main cooking fuel and around 16 percent of the wives in the household have finished at least primary education. The average age of the head of the household is around 41 years, while the average household size comprises 7 members. Only 17 percent are female headed households. Daily or weekly smoking is common in 21 percent of the households. Children’s gender is split equally between boys and girls, 75 percent had at least one vaccination and 41 percent are aged 0-1 years and 49 percent aged 2-4 years (for all values see S4 Table).

### 5.2 Estimation results

Column 2 of Table 1 presents the marginal effects from a probit model without household characteristics but including dummies for the country, the year of data collection and if the interview was conducted during the rainy season as controls. The model demonstrates that households cooking outdoors had lower occurrence of children’s acute respiratory infections in the two weeks preceding the survey. With a highly significant estimate, cooking outdoors is associated with a decrease in ARI by 0.5 percentage points compared to cooking indoors. This corresponds to a difference of 9 percent in the occurrence of ARI of households that cook outdoors compared to households that cook indoors (Table 1, column 2). This difference increases when restricting the observations to children aged from 0-1 years. In this case I find a reduction of 0.9 percentage points, which is again significant at a 1 percent level in the probit model and corresponds to a difference of 11 percent in the occurrence of ARI (column 3 of Table 1). Further controlling for several socio-economic household characteristics, children characteristics and household fuels in columns 3 and 5 (Table 1) shows that the results are very stable and even increase slightly for children aged 0-1 to 1 percentage point.

**Table 1:** ARI occurrence in rural areas (probit results) in the last two weeks preceding the survey^a^^,^^b^.

	ARI Children 0-4 years *margins*	ARI Children 0-4 years *margins*	ARI Children 0-1 years *margins*	ARI Children 0-1 years *margins*
Outdoor cooking	-0.005*** (0.00)	-0.005*** (0.00)	-0.009*** (0.00)	-0.010*** (0.00)
Electricity as cooking fuel		-0.022*** (0.01)		-0.045*** (0.01)
Gas as cooking fuel		-0.014*** (0.00)		-0.032*** (0.01)
Kerosene as cooking fuel		-0.029*** (0.01)		-0.033*** (0.01)
Coal, Lignite / Charcoal as cooking fuel		-0.000 (0.00)		-0.003 (0.00)
Other cooking fuel		0.007 (0.00)		0.003 (0.00)
Child is female		-0.003*** (0.00)		-0.006*** (0.00)
Observations	219,776	218,970	90,747	90,416
Country dummies	Yes	Yes	Yes	Yes
Year of data collection dummies	Yes	Yes	Yes	Yes
Interview in rainy season dummy	Yes	Yes	Yes	Yes
Household characteristics	No	Yes	No	Yes

^a:^ *, **, *** indicate p-values at the 10 percent level, 5 percent level and 1 percent level, respectively. All estimations are clustered on household level and standard errors are in brackets. S5 Table gives the coefficients to the marginal effects. Using wood as cooking fuel is the baseline category for the variable on cooking fuels and outdoor cooking is analysed in respect to indoor cooking.

^b:^ DHS all country dataset from 2005-2014.

Electricity, gas, and kerosene turn out to have a negative association with the occurence of ARI in the last 2 weeks preceding the survey. This effect is as high as 2.1 percentage points for electricity or 2.2 percentage points for kerosene. There is no difference in ARI occurrence between young children exposed to coal and those exposed to firewood as a fuel source (see Table 1, column 3). The coefficients are very high but as only few households are cooking with kerosene, electricity or gas the point estimates should be treated with caution.

**Fig 1:**
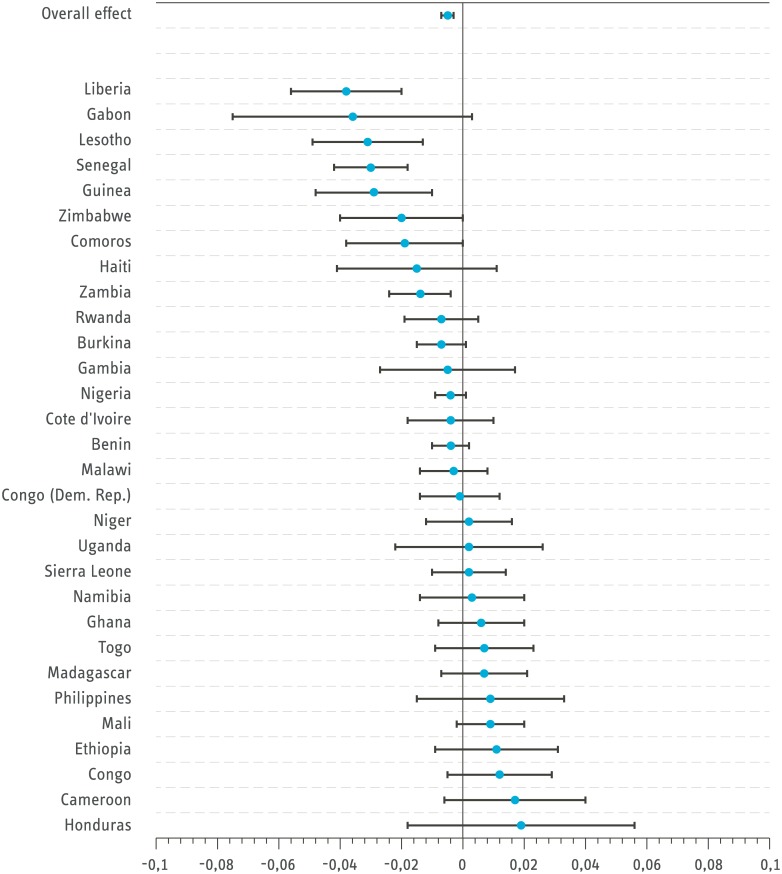
Effect size of outdoor cooking on ARI for children aged 0-4 (Probit estimation with controls). Bars signal 95 percent confidence intervals. Standard errors are clustered on household level. Congo refers to the Republic of Congo and Congo (Dem. Rep) refers to the Democratic Republic of the Congo. *Source: DHS all country dataset from 2005-2014.*

Fig 1 presents estimates of the mean effect of outdoor cooking on ARI based on models estimated for all countries separately. Among the 18 countries with a negative association between outdoor cooking and ARI, the effects of 7 are statistically significant. Otherwise, the country estimates are statistically insignificant.

**Fig 2:**
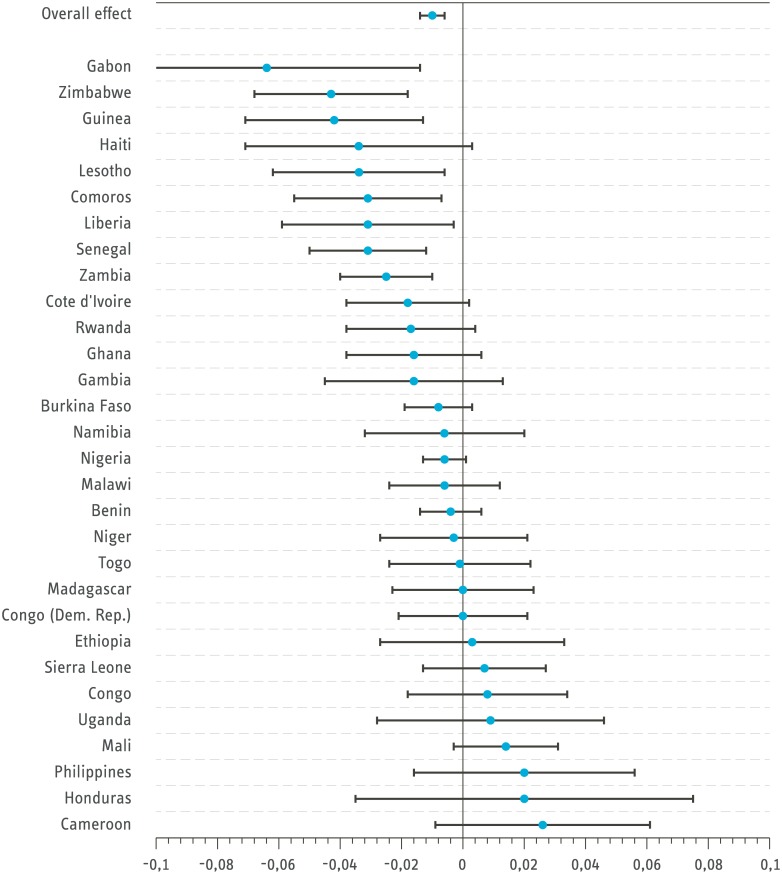
Effect size of outdoor cooking on ARI for children aged 0-1 (Probit estimation with controls). Bars signal 95 percent confidence intervals. Standard errors are clustered on household level. Congo refers to the Republic of Congo and Congo (Dem. Rep) refers to the Democratic Republic of the Congo. *Source: DHS all country dataset from 2005-2014.*

The results are similar but more pronounced when reducing the sample to children aged between 0 and 1 only (see Fig 2). In this case, 20 countries exhibit a negative correlation between outdoor cooking and ARI. Of these, 10 of the estimated effects are statistically significant. There is again no country with a significantly positive relationship between ARI and outdoor cooking.

Although the general trend shows a negative relationship between outdoor cooking and ARI, the relationship on the country level is heterogeneous. It is not entirely clear why this is the case, but the following provides some reasoning for the result. In some countries, many households have fixed built-in versions of cookstoves. In most of the Sub-Saharan African countries, portable stoves that can be used for cooking both inside and outside are prominent but some countries like Ethiopia and Uganda have a long tradition of using built-in stoves. With built-in stoves, households cook indoors but the emission in the cooking process is reduced. Thus, the particulate matter concentration may be less indoors than cooking outdoors without a stove [6]. Built-in stoves are also popular in Honduras, where clean cooking solutions for stationary stoves have received large political support [6]. In a next step, the robustness of the results is assessed by limiting the dataset to firewood-using households only, and excluding households that cook in a separate building (see Table 2 and S6 and S7 Tables).

**Table 2:** ARI in the last 2 weeks preceding the survey in rural areas limited to firewood cooking households and excluding separate building cooking households ^a^^,^ ^b^.

	ARI Children 0-4 years *margins*	ARI Children 0-1 years *margins*	ARI Children 0-4 years *margins*	ARI Children 0-1 years *margins*
	**Firewood using households only**		**Seperate building households excluded**	
Outdoor cooking	-0.006*** (0.00)	-0.011*** (0.00)	-0.009*** (0.00)	-0.013*** (0.00)
Observations	203,068	83,440	135,542	56,141
Country dummies	Yes	Yes	Yes	Yes
Year of data collection dummies	Yes	Yes	Yes	Yes
Interview in rainy season dummy	Yes	Yes	Yes	Yes
Household characteristics	Yes	Yes	Yes	Yes

^a:^ *, **, *** indicate p-values of a 10 percent level, 5 percent level and 1 percent level, respectively. All estimations are clustered on household level and standard errors are in brackets. S6 and S7 Tables give the coefficients to the marginal effects.

^b:^ DHS all country dataset from 2005-2014.

Dropping all households that do not use firewood as the main cooking fuel reduces the sample to 203,068 households. Estimating a probit model yields a larger negative coefficient, providing further evidence for the negative relationship between outdoor cooking and ARI among children (Table 2). S6 Table shows the full model. For households using firewood as the main cooking fuel, the negative association increases from -0.5 percentage points to -0.6 percentage points in the model with all controls (Table 2, column 1). Among children aged 0-1 the model shows an increase in the outdoor cooking coefficient from -1 percentage point to -1.1 percentage point. All relevant estimates remained statistically significant on a 1 percent level. The two principal models are re-estimated with only the households that cook in the house or outdoors, thereby excluding the households that cook in a separate building. The respective coefficients increase in size compared to the same estimations that included households cooking in separate building. Children aged 0-4 (0-1) living in households that cook outdoors suffered a -0.9 (-1.3) percentage point lower ARI incidence than those who live in households that cook indoors in the main house (see Table 2, column 3 and 4).

All models are further estimated using a matching approach with nearest neighbour matching without replacement. The marginal effects and significance levels are similar to those obtained using probit methods (see S8 Table for the full model). As a last robustness check, I estimate the principal models with the frequency of smoking in the household and vaccination of children as control variables. However, as both variables have large number of missing observations, it is not possible to include the two of them in the main specifications. In fact, it is even not possible to have both as control variables in the same regression as this would result in the dropping of around 75 percent of the observations. S9 Table reports the results on including frequency of smoking in the household as well as vaccination status of children separately. With the caveat of a largely reduced sample in mind, the results including vaccination as control variable are a bit lower but in general very similar to the main results. Those including frequency of smoking in the households as additional control yield an increased negative coefficient of -1.6 percentage points for children aged 0-1. For this sample, a reduction of -1.6 percentage points is corresponding to a decrease of 27 percent of ARI among children aged 0-1.

## 6 Conclusion

This paper analyzes the relationship between outdoor cooking and children’s acute respiratory infections in 30 developing countries over 3 continents. The results demonstrate a large and statistically significant relationship between outdoor cooking and the occurrence of ARI with an emphasis on children aged 0-1.

Although it was not possible to definitely isolate a causal relationship, the results point to the fact that outdoor cooking has a substantial association with children’s health. It is calculated that the effect size is as large as 9 percent for children between 0 and 4 when controlling for potential confounders. The effect size is even larger for children aged between 0 and 1, reaching a magnitude of 13 percent. This larger effect size for the very young children is attributed to the fact that the childrens’ body is more susceptible to particulate smoke emission at this age and children are more likely to be with their mothers. Estimating the main specifications excluding those households that cook in a separate building yields a larger coefficient on outdoor cooking compared to in-house cooking only. A last robustness check, including smoking frequency in the households, shows that the effect size increases starkly to -1.6 percentage points when controlling for this confounder for the children aged 0-1.

The overall results are complementary to those found by Buchner and Rehfuess (2015) and Rehfuess et al. (2009), as both find a significant negative association between outdoor cooking and ARI. This paper extends the analysis not only by including more countries but also by exploiting a large degree of heterogeneity between countries with respect to the incidence of cooking outdoors and the associated impact on children’s health.

Several caveats of the analysis point to areas where future research is needed: The dataset does not contain information on the type of cooking stoves used, fuel stacking, or other potential cofounders, making it impossible to assess the impact of those factors on ARI. The analysis thus rests on the assumption that any omitted variable is not systematic and does not bias the results extensively. I also have no information on the children’s presence at the cooking site. It is likely that the direct effect on childrens’ health is higher if all children remain in close proximity to the cooking site as suggested by studies that only examine the particulate emission ([38], for example). This may partly explain the difference of the magnitude in the effects for children aged 0-1 and children aged 0-4. However, this approach has the virtue of exploiting the occurrence of ARI, compared to other studies that examine the emittance of particulate emission only.

In this respect the results can be related to the influence of ARI on child mortality. Smith et al. (2014) report that around 466,000 children died in 2010 due to respiratory infections [43]. Assuming that decreases in deaths from ARI are proportional to decreases in its incidence, the estimated 9 percent reduction in ARI from cooking outdoors implies that 41,900 fewer children would die annually if everyone cooked outdoors.

The results of this study fit in the general literature on health hazards from particulate emission by significantly contributing to the scarce evidence of the impact of outdoor cooking on health hazards. Several policy implications emerge. Most importantly, the results demonstrate that simple behavioral interventions, such as promoting outdoor cooking, can have a substantial impact on health hazards. Of course, not everyone can cook outside the whole year due to unfavorable seasonal conditions. Such constraints should be taken into account when formulating policy, targeting behavioral interventions in those regions where there is scope for moving the cooking location outdoors. With regards to child protection, raising awareness of the detrimental effects of smoke emission on children and the benefits of leaving the children only a few meters away from the cooking place is important, particularly in countries where young children are always with their mothers. Behavioral interventions are nevertheless no panacea and should be complemented by the distribution of clean cookstoves. Yet, adopting cleaner fuels, such as LPG, has proven to be difficult and will be a long-term goal. In the meantime, policy effort should concentrate on promoting behavioral strategies and ventilation, with the extreme case of outdoor cooking being an important component.

In this regard, more solid evidence on the influence of different behavioral factors such as ventilation practices, outdoor cooking or general cooking behavior on health issues in general and children’s health in particular is needed. The combination of strategies targeting cookstove adoption and low-cost health knowledge interventions is a promising path for policy makers. Applying state of the art techniques, such as randomized controlled trials, to evaluate such interventions would be beneficial.

## Supporting information

S1 Table Sample description of households in rural areas *Source:* DHS all country dataset from 2005–2014. (PDF)

S2 Table Description of variables included in the analysis. (PDF)

S3 Table Outdoor cooking and acute respiratory infections (ARI) prevalence for households in rural areas *Source:* DHS all country dataset from 2005–2014. (PDF)

S4 Table Descriptive statistics *Source:* DHS all country dataset from 2005–2014. (PDF)

S5 Table Probit estimation of ARI in rural areas with coefficients and marginal effects *, **, *** indicate p-values at the 10 percent level, 5 percent level and 1 percent level, respectively. All estimations are clustered on the household level and standard errors are in brackets. *Source*: DHS all country dataset from 2005–2014.
(PDF)

S6 Table Probit estimation of ARI in rural areas with coefficients and marginal effects for firewood using households only *, **, *** indicate p-values at the 10 percent level, 5 percent level and 1 percent level, respectively. All estimations are clustered on the household level and standard errors are in brackets. *Source*: DHS all country dataset from 2005–2014.
(PDF)

S7 Table Probit estimation of ARI for households cooking in the main house or outdoors in rural areas with coefficients and marginal effects *, **, *** indicate p-values at the 10 percent level, 5 percent level and 1 percent level, respectively. All estimations are clustered on the household level and standard errors are in brackets. *Source*: DHS all country dataset from 2005–2014.
(PDF)

S8 Table Estimation of ARI in rural areas with coefficients and marginal effects (matched results) *, **, *** indicate p-values at the 10 percent level, 5 percent level and 1 percent level, respectively. All estimations are clustered on the household level and standard errors are in brackets. *Source*: DHS all country dataset from 2005–2014.
(PDF)

S9 Table Estimation of ARI in rural areas with coefficients and marginal effects with vaccination and frequency of smoking as further control variables *, **, *** indicate p-values at the 10 percent level, 5 percent level and 1 percent level, respectively. All estimations are clustered on the household level and standard errors are in brackets. *Source*: DHS all country dataset from 2005–2014.
(PDF)
