# Supplementary material for: Firewood, smoke and respiratory diseases in developing countries—The neglected role of outdoor cooking
Source: PLoS One. 2017 Jun 28;12(6):e0178631. doi: 10.1371/journal.pone.0178631 (PMC5489158; doi:10.1371/journal.pone.0178631)
Supplement: S1 Table — (PDF) [file pone.0178631.s001.pdf]

Table 1: Sample description of households in rural areas

**Country**	**Continent(Region)**	**Survey year(s)**	**Observations**
Benin	Africa(West)	2012	7,450
Burkina Faso	Africa (West)	2010	9,759
Cameroon	Africa (Central/South)	2011	4,454
Comoros	Africa (East)	2012	1,672
Cote d'Ivoire	Africa (West)	2012	3,777
Democratic Republic of the Congo	Africa (Central/South)	2014	9,211
Ethiopia	Africa (East)	2011	7,731
Gabon	Africa (Central/South)	2012	1,485
Gambia	Africa (West)	2013	4,600
Ghana	Africa (West)	2008	4,622
Guinea	Africa (West)	2012	3,841
Haiti	Latin America	2006,2012	7,156
Honduras	Latin America	2012	5,110
Lesotho	Africa (Central/South)	2009,2014	3,848
Liberia	Africa (West)	2013	6,099
Madagascar	Africa (East)	2009	8,672
Malawi	Africa (East)	2010	12,791
Mali	Africa (West)	2013	6,739
Namibia	Africa (Central/South)	2007,2013	4,280
Niger	Africa (West)	2012	8,030
Nigeria	Africa (West)	2006,2013	33,755
Philippines	Asia	2008,2013	5,659
Republic of the Congo	Africa (Central/South)	2012	5,118
Rwanda	Africa (East)	2010	10,480
Senegal	Africa (West)	2011	6,955
Sierra Leone	Africa (West)	2013	8,882
Togo	Africa (West)	2014	3,672
Uganda	Africa (East)	2006,2011	8,457
Zambia	Africa (East)	2007,2014	9,562
Zimbabwe	Africa (Central/South)	2005,2011	5,909
**Total**			219,776

Source: DHS all country dataset from 2005–2014.
