# Supplementary material for: Firewood, smoke and respiratory diseases in developing countries—The neglected role of outdoor cooking
Source: PLoS One. 2017 Jun 28;12(6):e0178631. doi: 10.1371/journal.pone.0178631 (PMC5489158; doi:10.1371/journal.pone.0178631)
Supplement: S2 Table — (PDF) [file pone.0178631.s002.pdf]

Table 2: Description of variables included in the analysis

	**Analysis level**
Acute respiratory infection in the last 2 weeks (constructed from the questions if children had coughing in the last two weeks preceeding the interview in combination with short, rapid breaths and problems in the chest), children gender, children vaccination	Children level
Type of cooking fuel used in the household, household cooking outdoors, age of head of households, head of household is female, number of household members, wife has at least primary education, year of the interview, house build out of high quality material (like stone), household owns a bike, household owns a motorcycle, household owns a car, household has a television, household has access to piped water, frequency of smoking in the household	Household Level
Rainy season	Regional level
Country dummies, interview year dummies	Country level
