# Supplementary material for: Firewood, smoke and respiratory diseases in developing countries—The neglected role of outdoor cooking
Source: PLoS One. 2017 Jun 28;12(6):e0178631. doi: 10.1371/journal.pone.0178631 (PMC5489158; doi:10.1371/journal.pone.0178631)
Supplement: S3 Table — (PDF) [file pone.0178631.s003.pdf]

Table 3: Outdoor cooking and acute respiratory infections (ARI) prevalence for households in rural areas

Country	Share of households cooking outdoors	Share of ARI prevalence	Share of ARI for children aged 0-1	Number of Observations of children aged 0-4 (of children aged 0-1)
**Africa**				
Burkina Faso	0.71 (0.45)	0.02 (0.13)	0.02(0.15)	9,759 (3,999)
Benin	0.59 (0.49)	0.01 (0.11)	0.02 (0.12)	7,450 (2,944)
Cameroon	0.24 (0.43)	0.08 (0.27)	0.10 (0.30)	4,454 (1,958)
Comoros	0.22 (0.41)	0.03 (0.18)	0.04 (0.19)	1,672 (690)
Cote d'Ivoire	0.49 (0.50)	0.04 (0.20)	0.05 (0.21)	3,777 (1,628)
Democratic Republic of the Congo	0.34 (0.47)	0.09 (0.29)	0.12 (0.33)	9,211 (3,697)
Ethiopia	0.15 (0.36)	0.08 (0.28)	0.10 (0.31)	7,731 (2,921)
Gabon	0.31 (0.46)	0.13 (0.33)	0.12 (0.32)	1,485 (644)
Ghana	0.46 (0.50)	0.06 (0.23)	0.06 (0.24)	4,622 (1,938)
Gambia	0.11 (0.32)	0.05 (0.21)	0.06 (0.24)	4,600 (2,092)
Guinea	0.34 (0.47)	0.08 (0.26)	0.10 (0.30)	3,841 (1,642)
Liberia	0.58 (0.49)	0.11 (0.32)	0.14 (0.35)	6,099 (2,512)
Lesotho	0.56 (0.50)	0.08 (0.27)	0.09 (0.28)	3,848 (1,721)
Madagascar	0.11 (0.32)	0.03 (0.17)	0.04 (0.20)	8,672 (3,428)
Mali	0.18 (0.39)	0.02 (0.14)	0.02 (0.14)	6,739 (2,714)
Malawi	0.29 (0.46)	0.09 (0.28)	0.10 (0.30)	12,791 (5,122)
Niger	0.81 (0.39)	0.05 (0.22)	0.07 (0.26)	8,030 (3,196)
Nigeria	0.23 (0.42)	0.03 (0.17)	0.04 (0.19)	33,755 (14,287)
Namibia	0.37 (0.48)	0.08 (0.26)	0.08 (0.27)	4,280 (1,903)
Republic of the Congo	0.46 (0.50)	0.06 (0.24)	0.08 (0.26)	5,118 (2,138)
Rwanda	0.19 (0.40)	0.06 (0.23)	0.08 (0.27)	10,480 (3,937)
Sierra Leone	0.58 (0.49)	0.07 (0.26)	0.09 (0.28)	8,882 (3,858)
Senegal	0.11 (0.32)	0.05 (0.22)	0.05 (0.23)	6,955 (2,918)
Togo	0.63 (0.48)	0.05 (0.22)	0.05 (0.22)	3,672 (1,536)
Uganda	0.21 (0.41)	0.23 (0.42)	0.27 (0.45)	8,457 (3,484)
Zambia	0.38 (0.48)	0.05 (0.23)	0.06 (0.25)	9,562 (3,937)
Zimbabwe	0.11 (0.32)	0.07 (0.26)	0.07 (0.26)	5,909 (2,617)
Overall Africa	0.34 (0.47)	0.06 (0.23)	0.08 (0.26)	201,851 (83,461)
**Asia**				
Philippines	0.15 (0.36)	0.09 (0.28)	0.09 (0.28)	5,659 (2,207)
Overall Asia	0.15 (0.36)	0.09 (0.28)	0.09 (0.28)	5,659 (2,207)
**Latin America**				
Honduras	0.12 (0.32)	0.18 (0.39)	0.19 (0.39)	5,110 (2,141)
Haiti	0.61 (0.49)	0.20 (0.40)	0.20 (0.40)	7,156 (2,938)
Overall Latin America	0.43 (0.49)	0.19 (0.39)	0.20 (0.40)	12,266 (5,079)
**Overall**	0.34 (0.47)	0.07 (0.25)	0.08 (0.27)	219,776 (90,747)

Note: Descriptive variables are weighted with official DHS survey weights. Standard deviations in brackets.
Source: DHS all country dataset from 2005–2014.
