# Supplementary material for: Firewood, smoke and respiratory diseases in developing countries—The neglected role of outdoor cooking
Source: PLoS One. 2017 Jun 28;12(6):e0178631. doi: 10.1371/journal.pone.0178631 (PMC5489158; doi:10.1371/journal.pone.0178631)
Supplement: S4 Table — (PDF) [file pone.0178631.s004.pdf]

Table 4: Descriptive statistics

	Observation	Mean
Household characteristics		
Share of households (in percent) cooking with	213,271	
Electricity	860	0.40
Gas	2,307	1.08
Firewood	196,241	92.01
Charcoal	10,337	4.85
Other	1,561	0.37
Kerosene	1,963	0.92
Wife has at least primary education (=1 if yes)	219,776	0.16 (0.37)
Household has access to piped water	219,643	0.17 (0.37)
House built out of high quality material (=1 if yes)	219,706	0.27 (0.44)
Age of head of household	219,685	40.75 (13.28)
Head of household is female (=1 if yes)	219,776	0.17 (0.37)
Number of household member	219,776	7.12 (4.15)
Household owns bike (=1 if yes)	219,698	0.33 (0.54)
Household owns motorcycle (=1 if yes)	219,678	0.17 (0.46)
Household owns car (=1 if yes)	219,705	0.03 (0.34)
Household owns television (=1 if yes)	219,738	0.14 (0.35)
Regular smoking in the house (=1 if yes)	122,391	0.21 (0.41)
Children characteristics		
Children aged 0-1 years (=1 if yes)	219,776	0.41 (0.49)
Gender of child (=1 if female)	219,776	0.50 (0.50)
Child has ever had a vaccination (=1 if yes)	111,959	0.75 (0.43)

Note: Descriptive variables are weighted with official DHS survey weights. Standard deviations in brackets.
Source: DHS all country dataset from 2005–2014.
