# Supplementary material for: Firewood, smoke and respiratory diseases in developing countries—The neglected role of outdoor cooking
Source: PLoS One. 2017 Jun 28;12(6):e0178631. doi: 10.1371/journal.pone.0178631 (PMC5489158; doi:10.1371/journal.pone.0178631)
Supplement: S7 Table — All estimations are clustered on the household level and standard errors are in brackets. Source: DHS all country dataset from 2005–2014. (PDF) [file pone.0178631.s007.pdf]

Table 7: Probit estimation of ARI for households cooking in the main house or outdoors in rural areas with coefficients and marginal effects

	**ARI** Children 0-4 years	**ARI** Children 0-4 years margins	**ARI** Children 0-4 years	**ARI** Children 0-4 years margins	**ARI** Children 0-1 years	**ARI** Children 0-1 years margins	**ARI** Children 0-1 years	**ARI** Children 0-1 years margins
Outdoor cooking	-0.067***	-0.008***	-0.076***	-0.009***	-0.083***	-0.011***	-0.097***	-0.013***
	(0.02)	(0.00)	(0.02)	(0.00)	(0.02)	(0.00)	(0.02)	(0.00)
Constant	9.789		8.121		2.055		-2.6888	
	(6.49)		(6.54)		(8.79)		(8.89)	
Observations	136,098	136,098	135,542	135,542	56,366	56,366	56,141	56,141
Country dummies	Yes	Yes	Yes	Yes	Yes	Yes	Yes	Yes
Year of data collection dummies	Yes	Yes	Yes	Yes	Yes	Yes	Yes	Yes
Interview in rainy season dummy	Yes	Yes	Yes	Yes	Yes	Yes	Yes	Yes
Household characteristics	No	No	Yes	Yes	No	No	Yes	Yes

Note: *, **, *** indicate p-values of a 10 percent level, 5 percent level and 1 percent level, respectively. All estimations are clustered on the household level and standard errors are in brackets.
Source: DHS all country dataset from 2005–2014.
