# Supplementary material for: Firewood, smoke and respiratory diseases in developing countries—The neglected role of outdoor cooking
Source: PLoS One. 2017 Jun 28;12(6):e0178631. doi: 10.1371/journal.pone.0178631 (PMC5489158; doi:10.1371/journal.pone.0178631)
Supplement: S8 Table — All estimations are clustered on the household level and standard errors are in brackets. Source: DHS all country dataset from 2005–2014. (PDF) [file pone.0178631.s008.pdf]

Table 8: Estimation of ARI in rural areas with coefficients and marginal effects (matched results)

	**ARI** Children 0-4 years	**ARI** Children 0-4 years margins	**ARI** Children 0-4 years	**ARI** Children 0-4 years margins	**ARI** Children 0-1 years	**ARI** Children 0-1 years margins	**ARI** Children 0-1 years	**ARI** Children 0-1 years margins
Outdoor cooking	-0.037***	-0.005***	-0.044***	-0.005***	-0.067***	-0.009***	-0.075***	-0.011***
	(0.01)	(0.00)	(0.01)	(0.00)	(0.02)	(0.00)	(0.02)	(0.00)
Constant	8.756		2.074		-6.711		-8.958	
	(5.93)		(6.09)		(8.13)		(8.20)	
Observations	147,637	147,637	147,498	147,498	61,148	61,148	61,090	61,090
Country dummies	Yes	Yes	Yes	Yes	Yes	Yes	Yes	Yes
Year of data collection dummies	Yes	Yes	Yes	Yes	Yes	Yes	Yes	Yes
Interview in rainy season dummy	Yes	Yes	Yes	Yes	Yes	Yes	Yes	Yes
Household characteristics	No	No	Yes	Yes	No	No	Yes	Yes

Note: *, **, *** indicate p-values of a 10 percent level, 5 percent level and 1 percent level, respectively. All estimations are clustered on the household level and standard errors are in brackets.
Source: DHS all country dataset from 2005–2014.
