# Supplementary material for: Firewood, smoke and respiratory diseases in developing countries—The neglected role of outdoor cooking
Source: PLoS One. 2017 Jun 28;12(6):e0178631. doi: 10.1371/journal.pone.0178631 (PMC5489158; doi:10.1371/journal.pone.0178631)
Supplement: S9 Table — All estimations are clustered on the household level and standard errors are in brackets. Source: DHS all country dataset from 2005–2014. (PDF) [file pone.0178631.s009.pdf]

Table 7: Estimation of ARI in rural areas with coefficients and marginal effects with vaccination and frequency of smoking as further control variables

	**ARI** Children 0-4 years	**ARI** Children 0-4 years margins	**ARI** Children 0-1 years	**ARI** Children 0-1 years margins	**ARI** Children 0-4 years	**ARI** Children 0-4 years margins	**ARI** Children 0-1 years	**ARI** Children 0-1 years margins
	**Regular smoking in the house**				**Child has received at least one vaccination**			
Outdoor cooking	-0.083***	-0.009***	-0.126***	-0.016***	-0.031**	-0.004**	-0.051**	-0.007**
	(0.01)	(0.00)	(0.02)	(0.00)	(0.01)	(0.00)	(0.02)	(0.00)
Observations	122,380	122,380	50,313	50,313	111,945	111,945	38,012	38,012
Country dummies	Yes	Yes	Yes	Yes	Yes	Yes	Yes	Yes
Year of data collection dummies	Yes	Yes	Yes	Yes	Yes	Yes	Yes	Yes
Interview in rainy season dummy	Yes	Yes	Yes	Yes	Yes	Yes	Yes	Yes
Household characteristics	No	No	Yes	Yes	No	No	Yes	Yes
Regular smoking in house	Yes	Yes	Yes	Yes	No	No	No	No
Vaccination	No	No	No	No	Yes	Yes	Yes	Yes

Note: *, **, *** indicate p-values of a 10 percent level, 5 percent level and 1 percent level, respectively. All estimations are clustered on the household level and standard errors are in brackets.
Source: DHS all country dataset from 2005–2014.
